# CDC Grand Rounds: Preventing Unsafe Injection Practices in the U.S. Health-Care System

**Published:** 2013-05-31

**Authors:** Guthrie Birkhead, Thomas E. Hamilton, Rachel Kossover, Joseph Perz, Denise Gangadharan, John Iskander

**Affiliations:** Office of Public Health, New York State Dept of Health; Survey and Certification Group, Center for Clinical Standards and Quality, Centers for Medicare & Medicaid Svcs; Div of Healthcare Quality Promotion; Office of the Director, CDC

## Background

Injectable medicines commonly are used in health-care settings for the prevention, diagnosis, and treatment of various illnesses. Examples include chemotherapy, intravenous antibiotics, vaccinations, and medications used for sedation and anesthesia. Medical injections often are administered in conjunction with surgical procedures, endoscopy, imaging studies, pain control, and cosmetic or complementary and alternative medicine procedures. Safe manufacturing and pharmacy practices are essential because every injection must begin with sterile medication. The appropriate medication must then be safely prepared (typically drawn up in a syringe), then administered in a manner that maintains sterility and minimizes risk for infection. Safe administration depends on adherence to the practices outlined in CDC’s evidence-based Standard Precautions guideline (1). Health-care providers should never 1) administer medications from the same syringe to more than one patient, 2) enter a vial with a used syringe or needle, or 3) administer medications from single-dose vials to multiple patients. They also should maintain aseptic technique at all times and properly dispose of used injection equipment.

## Scope of the Problem

Traditionally, injection safety has been recognized as a public health issue mainly in low- and middle-income country settings. Estimates of the global burden of disease associated with unsafe injections in the year 2000 included approximately 20 million new hepatitis B virus (HBV) infections, 2 million new hepatitis C virus (HCV) infections, and 250,000 new human immunodeficiency virus (HIV) infections (2). The U.S. experience with outbreaks attributed to unsafe injection practices has grown substantially over recent years. Since 2001, at least 49 outbreaks have occurred because of extrinsic contamination of injectable medical products at the point of administration (3; CDC, unpublished data, 2013). Twenty-one of these outbreaks involved transmission of HBV or HCV; the other 28 represented outbreaks of bacterial infections, primarily invasive bloodstream infections. Approximately 90% of these known outbreaks occurred in outpatient settings. Pain management clinics, where injections often are administered into the spine and other sterile spaces using preservative-free medications, and cancer clinics, which typically provide chemotherapy or other infusion services to patients who might be immunocompromised, are represented disproportionately relative to the overall volume of outpatient care.

Although hundreds of patients became infected in the outbreaks described, there is the additional burden of the estimated 150,000 patients during 2001–2012 who required notification advising them to undergo bloodborne pathogen testing after their potential exposure to unsafe injections (3; CDC, unpublished data, 2013).

Unsafe injection practices fall into two overlapping categories: reuse of syringes and mishandling of medications. “Direct” syringe reuse occurs when a single syringe is used for more than one person, as when the same syringe is used to inject via intravenous tubing or only the needle is changed between patients. These unsafe practices are still encountered; recently, several large patient notification events have stemmed from reuse of insulin injection pens for multiple patients (3,4). There is also growing recognition of provider-to-patient HCV transmission in the context of narcotics theft. In these scenarios, HCV infection is transmitted to patients as a consequence of overt syringe reuse (after the HCV-infected health-care provider had self-injected) or from contamination of medication that was accessed with a used syringe. Outbreaks involving infected health-care providers who obtained injectable drugs illicitly have affected large numbers of patients (5). “Indirect” syringe reuse (i.e., accessing shared medication vials with a used syringe) often is identified during outbreak investigations. Mishandling of medications primarily involves reuse of single-dose vials, which are intended for single-patient use only, to obtain medication for multiple patients. Because single-dose vials typically lack preservatives, this practice carries substantial risks for bacterial contamination, growth, and infection. Similarly, intravenous solution bags often are mishandled, for example, when inappropriately used as a common source of supply for multiple patients.

## Case Study

Outbreaks investigated by CDC and state and local health departments have illustrated that the U.S. health-care system is susceptible to the dangers of unsafe injections. The investigation of an HCV outbreak in Nevada in 2008 revealed that reuse of syringes on multiple patients and use of single-use medication vials on multiple patients was the likely mechanism by which HCV infections were transmitted (6). The ambulatory surgical center (ASC) under investigation used the sedative, propofol, which is supplied in single-dose vials, during endoscopy procedures (Figure). At the start of a procedure, a new, clean needle and syringe were used to draw up medication. When used on an HCV-infected patient, backflow contaminated the syringe. Patients typically required additional medication to maintain sedation, and instead of using a new needle and syringe, nurses in this clinic reused the patient’s syringe to draw up this medication, after replacing the needle. By putting the reused syringe in contact with the vial, contamination was transferred to the vial. This clinic routinely reused these single-dose vials for multiple patients, which established a pathway for the spread of HCV from one patient to another. Changing the needle in this situation did not prevent contamination of the vial; however, it did expose the nurse to the risk for a sharps injury and occupational disease transmission. To avoid this risk, a new needle and syringe should be used every time a vial is accessed to withdraw medication.

## State and Federal Responses

Several states are addressing the public health issue of unsafe injection practices, including New York. Since 2002, the New York State Department of Health (NYSDOH) has conducted 11 investigations of known or potential bloodborne pathogen transmissions that involved notification of nearly 10,000 persons (NYSDOH, unpublished data, 2012). The predominant modes of exposure or transmission discovered were related to unsafe injection practices similar to those described in the Nevada outbreak. NYSDOH also has implemented policy and educational initiatives as part of a comprehensive public health response to the investigations. These include 1) changes to the public health law in 2008 to strengthen the ability of the health department to investigate and hold physicians accountable for poor infection control practices and to update infection control and barrier precautions training mandated by NYSDOH (7), and 2) partnering with CDC on the One & Only campaign, a health-care provider and public education campaign targeting injection safety (4).

The Centers for Medicaid & Medicare Services (CMS), the single largest purchaser of health care in the United States, seeks to promote innovation and the consistent advancement of safety and quality of health care. Many, but not all, types of facilities that participate in Medicare or Medicaid are subject to unannounced, onsite inspections by state or federal surveyors to be certified under those programs. Examples of such regulated facilities are ASCs, clinical laboratories, dialysis facilities, hospitals, and nursing homes (8). ASCs are one of the fastest growing types of facility among Medicare-participating providers and suppliers. Characteristics of ASCs, such as the large number of facilities and the variety of their size, scope, and complexity of practice, make them particularly challenging settings for government oversight to ensure proper infection control procedures. Physician offices and specialty clinics that do not seek CMS status as a certified ASC typically are not subject to survey and certification.

CDC, CMS, and the state of Nevada began an intense collaboration during the 2008 HCV outbreak (6) investigation. CMS strengthened the requirements for infection control to require that ASCs maintain ongoing infection control programs, adhere to professional standards (1), designate a qualified infection control professional, and implement nationally recognized infection control guidelines. Out of the experience in Nevada, a worksheet that CMS surveyors could use to better identify lapses in infection control, including injection safety, was developed.* The effectiveness of the worksheet in identifying infection control lapses was tested in a pilot study involving three volunteer states (Maryland, North Carolina, and Oklahoma). Of the ASCs surveyed in these states, 67.6% had infection control lapses, 57.4% were cited for some type of deficiency in meeting CMS infection control requirements, and 29.4% were cited specifically for deficiencies in medication usage (e.g., multipatient use of single-dose medication vials was identified in 28.1% of ASCs) (9). Results from surveys of a randomly selected national sample of ASCs in 2010 showed that the findings from the pilot study could be generalizable to the rest of the country (CMS, unpublished data, 2012). Although recent data from surveys from the national sample of randomly selected ASCs reveal some improvements (with 51.3% of surveyed ASCs being cited for CMS deficiencies in infection control in 2010 nationally versus 43.5% in 2011), the overall national risk profile was very similar to the risks identified in the 2008 three-state pilot study (CMS, unpublished data, 2012).

## The Role of Public Health in Addressing Gaps in Injection Safety

Injection safety is a complex public health issue that requires a multidimensional approach. The four “E’s” for ensuring safe injections include 1) epidemiologic surveillance, reporting, monitoring, and investigation of outbreaks potentially related to unsafe injections; 2) educational initiatives to promote understanding and use of safe injection and basic infection control practices; 3) enforcement and oversight by federal and state authorities; and 4) engineering of devices, equipment, and processes to reduce or eliminate disease transmission risks.

Since 2009, CDC has worked to improve epidemiologic capacities at state health departments by supporting the formation and development of state programs that address health-care–associated infections (10). To bridge the education gap, CDC and its partners in the Safe Injection Practices Coalition developed the One & Only campaign. CDC’s Standard Precautions form the basis for the One & Only campaign’s messages. The ultimate goal of the campaign is to prevent outbreaks, infections, and the need for patient notification (4). Recognizing that education is necessary but not always sufficient, policies and mechanisms must be in place to 1) support and ensure that injection safety and infection control procedures are followed, and 2) mandate corrective action. Examples of proposed engineering solutions aimed at preventing syringe reuse include the redesign of syringes to change color after use or the incorporation of tamper-evident packaging. Implementation of the four “E’s” should help minimize unsafe injections practices; however, the One & Only campaign encourages patients to ask their health-care provider about bloodborne pathogen safety as part of increased patient involvement in medical decision making (4).

Unsafe injection practices put patients at risk for infection and have been associated with various procedures and settings. Unsafe injections also increase the financial and emotional burden borne by patients, health-care providers, and public health and medical-care systems. This harm is entirely preventable. To eliminate the problem of unsafe injections, injection safety interventions need to be implemented in all settings where injections are delivered. Many outpatient facilities, including oncology clinics, pain management clinics, and physician offices, typically do not fall within the purview of federal and state regulatory oversight of health-care facilities, thus making it difficult to monitor injection safety and other infection control practices. Unsafe injection practices have resulted in disease transmission and the need for notification of hundreds of thousands of patients. The risks of unsafe injections practices are unacceptable. The goal of public health and health-care systems should be to eliminate such risks immediately and definitively through comprehensive preventive actions.

## Figures and Tables

**FIGURE:**
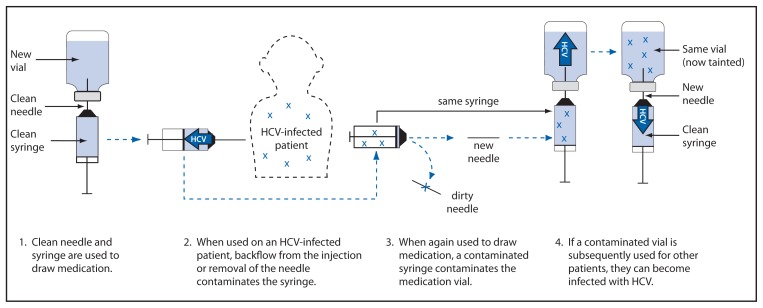
Unsafe injection practices and circumstances that likely resulted in transmission of hepatitis C virus (HCV) at a clinic — Las Vegas, Nevada, 2007 Source: CDC. Acute hepatitis C virus infections attributed to unsafe injection practices at an endoscopy clinic—Nevada, 2007. MMWR 2008;57:513–7. Available at http://www.cdc.gov/mmwr/preview/mmwrhtml/mm5719a2.htm.

## Footnotes

* Additional information available at http://www.cms.gov/surveycertificationgeninfo/downloads/scletter09_37.pdf.
